# Evaluating the U.S. Air Quality Index as a risk communication tool: Comparing associations of index values with respiratory morbidity among adults in California

**DOI:** 10.1371/journal.pone.0242031

**Published:** 2020-11-17

**Authors:** Kevin R. Cromar, Marya Ghazipura, Laura A. Gladson, Lars Perlmutt

**Affiliations:** 1 Marron Institute of Urban Management, New York University, New York, New York, United States of America; 2 New York University School of Medicine, New York, New York, United States of America; Texas A&M University College Station, UNITED STATES

## Abstract

**Background:**

The Air Quality Index (AQI) in the United States is widely used to communicate daily air quality information to the public. While use of the AQI has led to reported changes in individual behaviors, such behavior modifications will only mitigate adverse health effects if AQI values are indicative of public health risks. Few studies have assessed the capability of the AQI to accurately predict respiratory morbidity risks.

**Methods and findings:**

In three major regions of California, Poisson generalized linear models were used to assess seasonal associations between 1,373,165 respiratory emergency department visits and short-term exposure to multiple metrics between 2012–2014, including: daily concentrations of NO_2_, O_3_, and PM_2.5_; the daily reported AQI; and a newly constructed health-based air quality index. AQI values were positively associated (average risk ratio = 1.03, 95% CI 1.02–1.04) during the cooler months of the year (November-February) in all three regions when the AQI was very highly correlated with PM_2.5_ (R^2^ ≥ 0.89). During the warm season (March-October) in the San Joaquin Valley region, neither AQI values nor the individual underlying air pollutants were associated with respiratory morbidity. Additionally, AQI values were not positively associated with respiratory morbidity in the Southern California region during the warm season, despite strong associations of the individual underlying air pollutants with respiratory morbidity; in contrast, health-based index values were observed to be significantly associated with respiratory morbidity as part of an applied policy analysis in this region, with a combined risk ratio of 1.02 (95% CI: 1.01–1.03).

**Conclusions:**

In regions where individual air pollutants are associated with respiratory morbidity, and during seasons with relatively simple air mixtures, the AQI can effectively serve as a risk communication tool for respiratory health risks. However, the predictive ability of the AQI and any other index is contingent upon the monitored values being representative of actual population exposures. Other approaches, such as health-based indices, may be needed in order to effectively communicate health risks of air pollution in regions and seasons with more complex air mixtures.

## Introduction

Since 1980, air pollution concentrations in the United States have steadily decreased [1], but thousands of excess deaths and tens of thousands of excess morbidities are still attributed to exposure each year. In California alone, an estimated 3,100 excess deaths and 7,731 excess morbidity cases are attributable annually to air pollution exceeding recommended standards [2]. Health risks associated with short-term exposure to outdoor air pollution are reported to the public by the U.S. Environmental Protection Agency (EPA) through the Air Quality Index (AQI), which has been used as a model for many other air quality indices around the world [3–5].

Each city in the United States with a population over 350,000 is required to report the AQI to the public using a federally prescribed method, including detailed guidance on how to calculate and communicate risk associated with index values [6]. The AQI uses a simple aggregation method to determine local air quality: while all five criteria pollutants are considered, only the single pollutant with the highest concentration relative to its national standard is used to calculate the daily index value. As such, the AQI essentially functions as a regulatory-based, single-pollutant index calibrated to the National Ambient Air Quality Standards (NAAQS). These standards are reviewed by the EPA every five years [7], and although not required [8], the index values have been updated with each NAAQS revision.

The AQI was designed to provide a uniform and simple method of reporting air pollution concentrations that exceed federal regulatory levels. The AQI also provides a ready-made platform to alert the public to extreme pollution episodes. The existing literature shows evidence of avoidance behavior in response to air quality alerts among both susceptible populations and individuals with no underlying health conditions [9–13]. However, the AQI was not specifically designed to act as a risk communication tool for acute health risks, even though it has been appropriated for this purpose. Only the single pollutant with the highest NAAQS-related concentration determines the AQI daily value; thus, the potential additive or mixed effects of multiple pollutants on air quality are ignored [5, 14–16]. Since index levels are linked to health advisories, actual health risk may be understated or misrepresented using the existing AQI framework.

Alternatives to the AQI, and similarly constructed air pollution indices, have been developed in order to address the limitations of its inherent single-pollutant model [3–5, 17, 18]. The most mature efforts in this regard has been the development and implementation of an air quality health index (AQHI) in Canada [19]. In contrast to the U.S.-based AQI, in which index values reflect local air quality conditions, health-based index values go a step further in an effort to report the daily index values as a function of health risk. Health-based indices exist in several global nations, primarily modeled after the Canadian AQHI. The AQHI addresses many of the concerns surrounding the AQI, particularly by accounting for the multi-pollutant effects of air pollution when calculating health risk [19]. Health-based indices have been evaluated in Canada and China, where AQHI reports were found to generally reflect true health outcomes in study regions compared to existing single-pollutant, concentration-based models [14, 16, 20–22].

Despite the fact the AQI was not explicitly designed to function as a risk communication tool, it is possible that it may be able to functionally fulfill this objective. The present study tests the efficacy of the existing AQI in reflecting health risks by investigating associations between population-level health outcomes with index values. Specifically, associations between respiratory morbidity (emergency department (ED) visits) and daily AQI values and health-based index values were evaluated for California from 2012–2014. Due to its relatively poor air quality in the United States and its demographic and geographical diversity, California serves as an excellent case study for this analysis. Additionally, due to the aforementioned high excess morbidity and mortality attributable to air pollution, California is one of the regions in the United States that would most immediately benefit from a better understanding of the strengths and limitations of the AQI as a risk communication tool. However, the intent of this analysis is not to propose changes to California’s air quality reporting, but to use this location to test whether the current AQI is a generally reliable tool for reporting real health risks.

## Materials and methods

ED data was accessed via California’s Office of Statewide Health Planning and Development (data can be requested at https://oshpd.ca.gov/data-and-reports/research-data-request-information/). The study population was derived from adults over 18 years of age in 25 California counties, yielding a capture population of 32,708,938 residents constituting 86.9% of the state’s total population. All respiratory ED visits from 2012–2014 were gathered from this population and coded according to the 9th version of the International Classification of Diseases (ICD-9; World Health Organization, Geneva). Respiratory visits were defined as asthma (493), upper respiratory infection (465), pneumonia (480–486), and chronic obstructive pulmonary disease (490–496) (all 2-digit extensions were used). While the standards determining the AQI include mortality and cardiovascular disease in addition to respiratory morbidity, we chose to focus on respiratory health risks since these outcomes are most commonly used by the public in considering how to respond to health index reports [23].

Daily pollutant concentrations based on NAAQS averaging times (1-hour maximum for nitrogen dioxide [NO_2_], 8-hour maximum for ground-level ozone [O_3_], and 24-hour average for fine particulate matter [PM_2.5_]) and daily AQI values (retrospective, not forecast) were obtained from EPA Air Data at the county-level [24]. In counties with multiple monitors, the daily maximum and daily average from monitors with a threshold of 75% of available days per season was obtained. Meteorological data was collected for each county from January 2012 to December 2014, including daily temperature and relative humidity.

The daily health-based index used in this study was constructed using the quantitative approach used to develop the AQHI in Canada by Stieb et al. 2005 [15], and is meant to be a generic index that uses additive effects of nationally representative coefficients for PM_2.5_, O_3_, and NO_2_. These index values represent the additive excess risk of each individual pollutant on respiratory morbidity. The methods and individual studies used in the random effects meta-analysis to derive the coefficients for each pollutant are contained in S1 Appendix and S1 Table, respectively.

Time-series analysis using a quasi-Poisson generalized linear model (GLM) was used to determine the association between respiratory morbidity and index values. The quasi-likelihood estimation was used to account for overdispersion. Decisions in regard to model selection were made a priori based on epidemiology studies looking at the short-term effects of outdoor air pollution. The full GLM model incorporated the daily count of total respiratory ED visits, the daily pollutant concentration, and the daily AQI or health-based index value. Linear terms were used for day of the week, and non-linear adjustments were made using natural splines to control for long-term trends, seasonality, relative humidity, same day temperature, and the temperature at the average of lag days 1 to 3. Similar to the methods reported in other studies, the Akaike information criterion was used to select the optimal number of degrees of freedom (df), or knots, in the model [25–27]. Separate seasonal analyses were completed for the cooler months (November-February) and the warmer months (March-October). Analyses completed during the cool and warm seasons used 8 df and 16 df per season per year, respectively, to account for long-term trends and seasonality. This model was validated by comparing observed vs. fitted adjusted R^2^ values for each predictor variable by location and season (see S4 Table).

The associations between AQI values and pollutant concentrations with respiratory ED visits were assessed by relative risk (RR) and 95% confidence interval (CI) per inter-quartile range (IQR) increase in index values. County-level associations with AQI values or individual pollutant concentrations in both the warm and cool season were pooled using a random-effects model to account for heterogeneity between county estimates. Associations between newly constructed health-based index values were only completed for regions where AQI values were not significantly associated with population health risk. Primary results are reported for a 4-day average lag structure from lag days 0 through 3; results for all individual lag days investigated in the analysis are shown in S3 Table. Using county-level US Census data from 2012–2014 [28] a subsequent post-hoc analysis was conducted to determine if socioeconomic status impacted our results (results shown in S5 Table).

## Results

**Fig 1:**
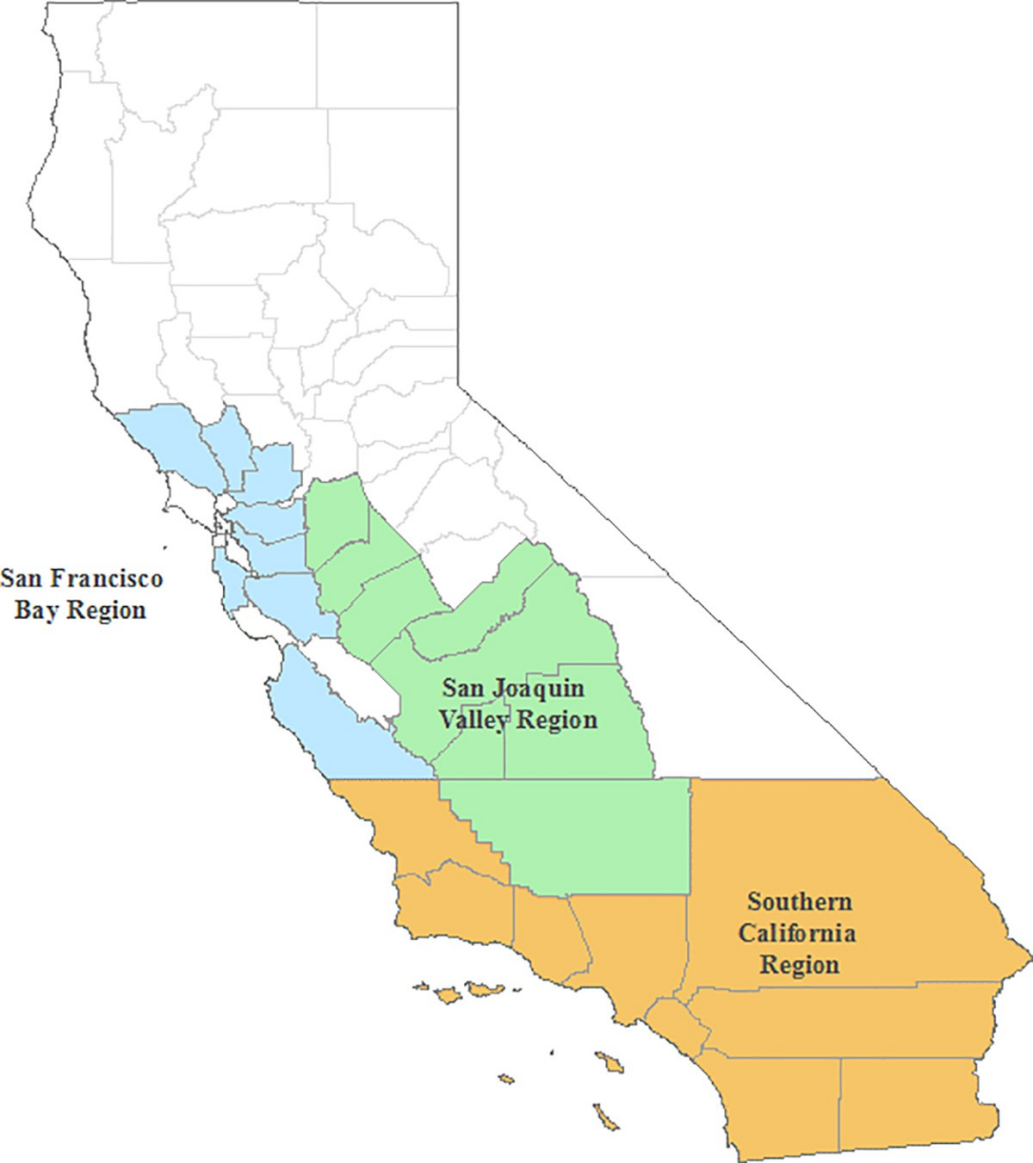
Map of regions and associated counties included in the study.

The study counties were grouped into three distinct regions based in part on geographic proximity, but primarily on similarity in air pollution profiles observed throughout the study period. Additionally, only counties that had both health information and monitor data for the three study pollutants were included, and as such represent areas where AQI values are fully informed by these criteria air pollutants. Fig 1 depicts a map of each region and their associated counties (see S2 Table for a breakdown of health events and pollutant concentrations for individual counties).

Demographics of respiratory ED visitors across all three years are presented in Table 1. Overall, 51.4% of visitors were over 40 years old and 60.4% were female. Race and ethnicity among visitors was 38% white, 35.7% Hispanic, 16.2% black, 4.3% Asian or Pacific Islander, and 0.3% Native American, Eskimo, or Aleut.

**Table 1:** Demographics of study population observed from 2012–2014 in three regions of California.

	San Francisco Bay Area	San Joaquin Valley	Southern California
**Total ED Visits**	300,228	233,715	839,222
**Age**			
18–40	139,227 (46.4%)	122,612 (52.5%)	405,300 (48.3%)
41–64	108,697 (36.2%)	78,940 (33.8%)	291,971 (34.8%)
65 +	52,304 (17.4%)	32,163 (13.8%)	141,951 (16.9%)
**Sex**			
Female	178,167 (59.3%)	143,170 (61.3%)	507,866 (60.5%)
Male	122,061 (40.7%)	90,545 (38.7%)	331,356 (39.5%)
**Race/Ethnicity**			
White	107,786 (35.9%)	96,773 (41.4%)	316,726 (37.7%)
Hispanic	84,217 (28.1%)	94,809 (40.6%)	311,663 (37.1%)
Black	63,211 (21.1%)	24,440 (10.5%)	134,765 (16.1%)
Asian / Pacific Islander	22,600 (7.5%)	4,378 (1.9%)	31,642 (3.8%)
Native American / Eskimo / Aleut	1,670 (0.6%)	650 (0.3%)	1,684 (0.2%)
Other	20,744 (6.9%)	12,665 (5.4%)	42,742 (5.1%)

Total ED visits over the study period are shown by age, sex, and race/ethnicity.

From 2012–2014, there were a total of 1,373,165 respiratory ED visits across the three study regions: Southern California (Ventura, Santa Barbara, San Louis Obispo, San Diego, San Bernardino, Riverside, Orange, Los Angeles, and Imperial counties) had 839,222 ED visits; San Joaquin Valley (Tulare, Stanislaus, San Joaquin, Merced, Madera, Kings, Kern, and Fresno counties) had 233,715 ED visits; and San Francisco Bay Area (Sonoma, Solano, Santa Clara, San Mateo, Napa, Monterey [not geographically in the San Francisco Bay area but its air quality profile matches the rest of the listed counties], Contra Costa, and Alameda counties) had 300,228 ED visits. As a result of the higher number of respiratory ED visits per day during the cooler season, the total number of counts was roughly equal between the two seasons, despite different numbers of days in each period, as shown in Table 2.

**Table 2:** Median and inter-quartile range for pollutant concentrations (24-hour PM_2.5_, 8-hour O_3_, and 1-hour NO_2_) and daily Air Quality Index, by region and season from 2012–2014.

Season	Region	Respiratory ED Visits		PM_2.5_ (μg/m^3^)		O_3_ (ppb)		NO_2_ (ppb)		AQI	
		*ED Visits per 100k people*	*Daily Avg*	*Median*	*IQR*	*Median*	*IQR*	*Median*	*IQR*	*Median*	*IQR*
**March—October**	Southern California	2,495	1,970	9.9	5.4	57	18	23	20.3	67	46
	San Joaquin Valley	3,283	532	9.2	5.8	58	21	19	15	68	52
	San Francisco Bay Area	2,709	720	6.9	4.8	38	12	13.9	13.3	40	15
**November—February**	Southern California	1,843	2,971	8.3	6.5	43	8	35.9	17.1	55	25
	San Joaquin Valley	2,601	861	17.6	18.4	34	13	28	16	71	47
	San Francisco Bay Area	1,904	1,033	9.5	8.4	30	11	28.7	13.6	43	25

Spearman correlations between AQI values, individual pollutant concentrations, and weather variables are shown by region and season in Table 3. Very high positive correlations (0.89 to 0.98) were observed for PM_2.5_ and AQI values in all three regions during the cooler months of the year; during the warmer months, there were only moderate correlations observed for PM_2.5_ and AQI values (0.58 to 0.71), in addition to moderate to high positive correlations for O_3_ and AQI values (0.66 to 0.89).

**Table 3:** Spearman correlations by season and region for air pollutants and meteorological variables, from 2012–2014.

		November–February						March–October					
Region	Variable	AQI	Health-based Index	PM_2.5_	NO_2_	O_3_	Temp	AQI	Health-based Index	PM_2.5_	NO_2_	O_3_	Temp
**Southern California**	Health-based Index	0.82						0.78					
	PM_2.5_	0.89	0.88					0.60	0.76				
	NO_2_	0.37	0.55	0.39				0.37	0.73	0.33			
	O_3_	0.27	0.40	0.15	0.07			0.89	0.80	0.48	0.42		
	Temp*	0.04	0.02	-0.01	0.06	0.08		0.38	0.28	0.28	0.23	0.39	
	RH**	-0.09	-0.24	-0.04	-0.46	-0.17	-0.23	-0.43	-0.47	-0.28	-0.52	-0.44	-0.62
**San Joaquin Valley**	Health-based Index	0.71						0.81					
	PM_2.5_	0.98	0.97					0.58	0.50				
	NO_2_	0.27	0.53	0.28				0.11	0.28	0.19			
	O_3_	0.30	0.23	0.28	0.20			0.69	0.60	0.38	0.13		
	Temp*	-0.17	-0.09	-0.18	-0.16	0.19		0.63	0.52	0.26	-0.04	0.42	
	RH**	-0.24	-0.14	-0.23	-0.20	-0.44	-0.10	-0.56	-0.66	-0.28	-0.05	-0.32	-0.67
**San Francisco Bay Area**	Health-based Index	0.79						0.80					
	PM_2.5_	0.97	0.95					0.71	0.70				
	NO_2_	0.53	0.63	0.54				0.38	0.50	0.24			
	O_3_	-0.14	-0.10	-0.18	0.01			0.66	0.77	0.31	0.05		
	Temp*	-0.30	-0.19	-0.34	-0.19	0.06		0.27	0.28	0.16	0.08	0.24	
	RH**	-0.19	-0.16	-0.16	-0.42	-0.26	0.05	-0.41	-0.45	-0.21	-0.31	-0.38	-0.48

* Mean temperature

** Mean relative humidity

### Individual pollutant results

**Fig 2:**
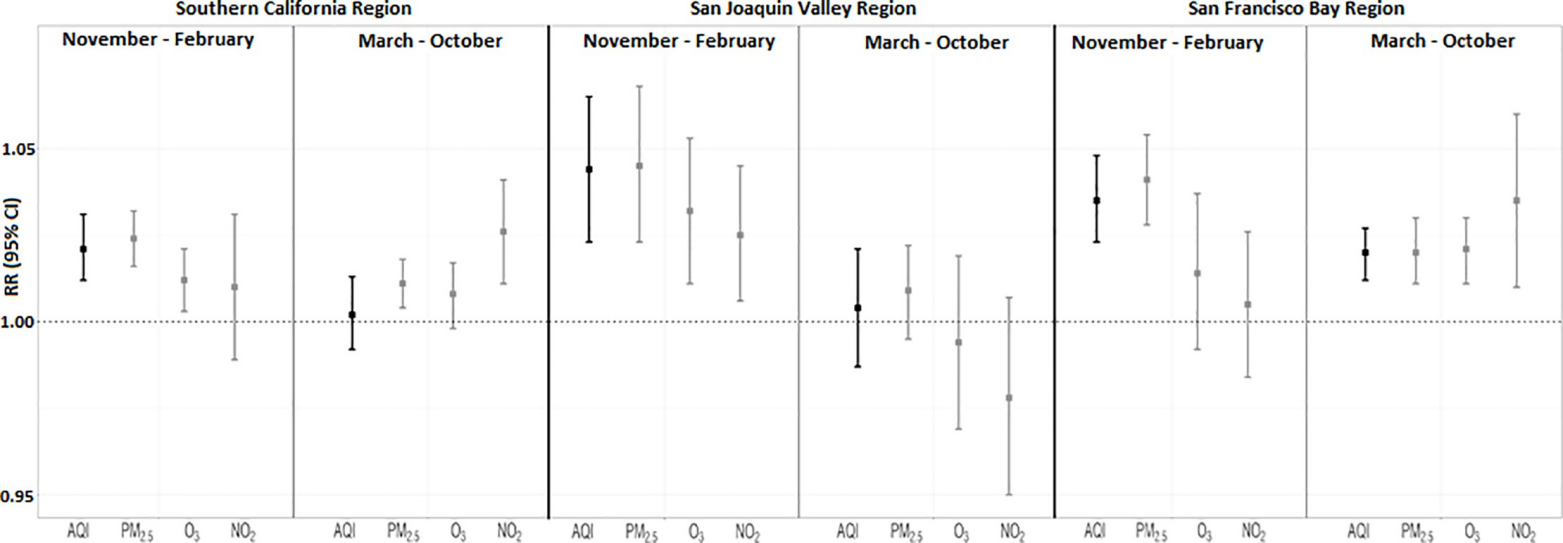
Relative risks of respiratory ED visits for AQI values and individual air pollutants for 2012–2014, by region. Relative risks are presented per IQR increase in AQI values and pollutant concentrations and are shown for the four-day moving average of lag days 0 through 3. Abbreviations: AQI, air quality index; CI, confidence interval; IQR, inter-quartile range; NO_2_, nitrogen dioxide; O_3_, ozone; RR, relative risk; PM_2.5_, fine particulate matter.

Fig 2 illustrates the association of individual air pollutants (PM_2.5_, NO_2_, and O_3_) with respiratory ED visits by season and region. The associations depicted in this figure are based on the IQR of the underlying pollutants, shown in Table 2. Of particular note is the lack of significant positive associations between the three individual pollutants and respiratory ED visits from March to October in the San Joaquin Valley region, even though positive significant associations were observed in this region during the cooler months. Also of note is the significant positive associations with morbidity risk for the individual pollutants in the Southern California region during the warmer months despite there being no significant association with AQI values during this same time period and region.

### AQI results

**Fig 3:**
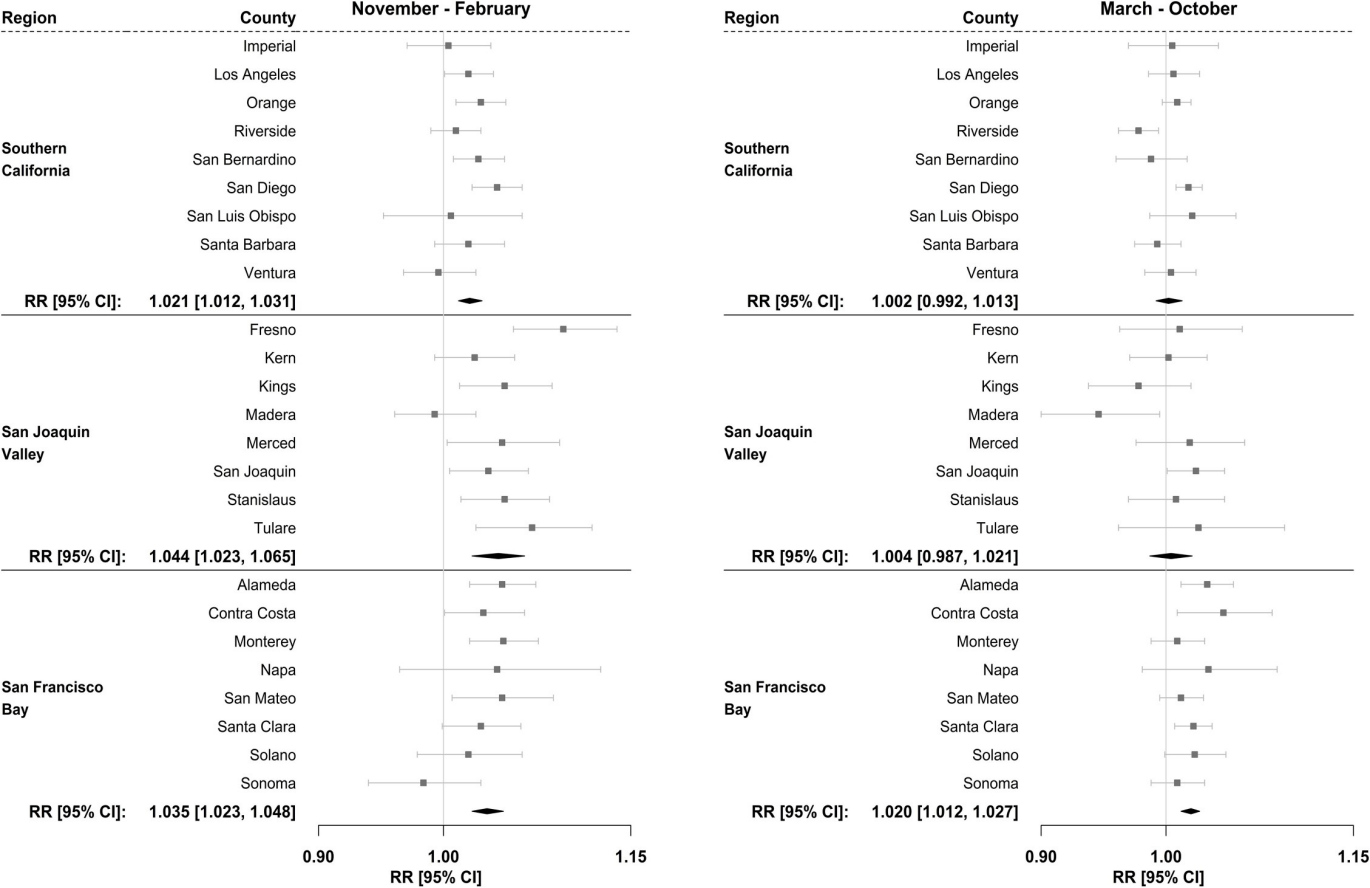
Relative risks of respiratory ED visits for inter-quartile change in AQI values during the cooler months (November to February) versus the warmer months (March to October) from 2012–2014, by county and region. Associations are shown for a four-day moving average of lag days 0 through 3. Abbreviations: AQI, air quality index; CI, confidence interval; RR, relative risk.

While there is some inter-county variation in observed associations, our pooled analysis found that the daily levels of the AQI are significantly associated with respiratory ED visits for all three study regions in the cooler months (November to February), as shown in Fig 3. An interquartile increase in AQI values was associated with a 4% increase in respiratory morbidity risk in San Joaquin Valley (RR: 1.04, 95% CI: 1.02–1.07 and San Francisco Bay Area (RR: 1.04, 95% CI: 1.02, 1.05), and with a 2% increase in respiratory morbidity in Southern California (RR: 1.02, 95% CI: 1.01, 1.03). More important than the magnitude of the relative risks is the observed positive associations of AQI values and population-level respiratory morbidity.

During the warmer months of the year (March to October), AQI values were significantly associated with respiratory ED visits in the San Francisco Bay region with a combined RR of 1.02 (95% CI: 1.01–1.03). All individual counties in this region showed positive associations, although not all positive associations were statistically significant at the county level.

However, unlike the San Francisco Bay Area, AQI values were not significantly associated with respiratory ED visits in either the San Joaquin Valley or the Southern California regions during the warm season. In Southern California, a significant positive association was only observed in San Diego County, offset by the significant negative association observed in Riverside County (RR: 0.98, 95% CI: 0.96–0.99) during the same time period. In the San Joaquin Valley region, none of the individual counties demonstrated a positive significant association between AQI and respiratory ED visits during the warmer months, and one county (Madera County) demonstrated significant negative associations (RR: 0.95, 95% CI: 0.90–0.99).

### Health-based index results

Due to the observed positive association of individual pollutants, but not AQI values, with respiratory morbidity in Southern California, an exploratory analysis was conducted to determine whether a health-based index combining the excess risk of three individual pollutants (PM_2.5_, O_3_, and NO_2_) would better predict population-level health risks in the region. In order to complete an applied policy analysis, the evaluation focused specifically on four counties comprising the Southcoast Air Quality Management District (Los Angeles, Orange, Riverside, and San Bernardino) in the Southern California region. Local air quality agencies are best suited for making specific decisions regarding risk communication, and therefore these four counties were selected for analysis in an effort to demonstrate how these types of assessments can be directly relevant to the management level where such decisions are made.

**Fig 4:**
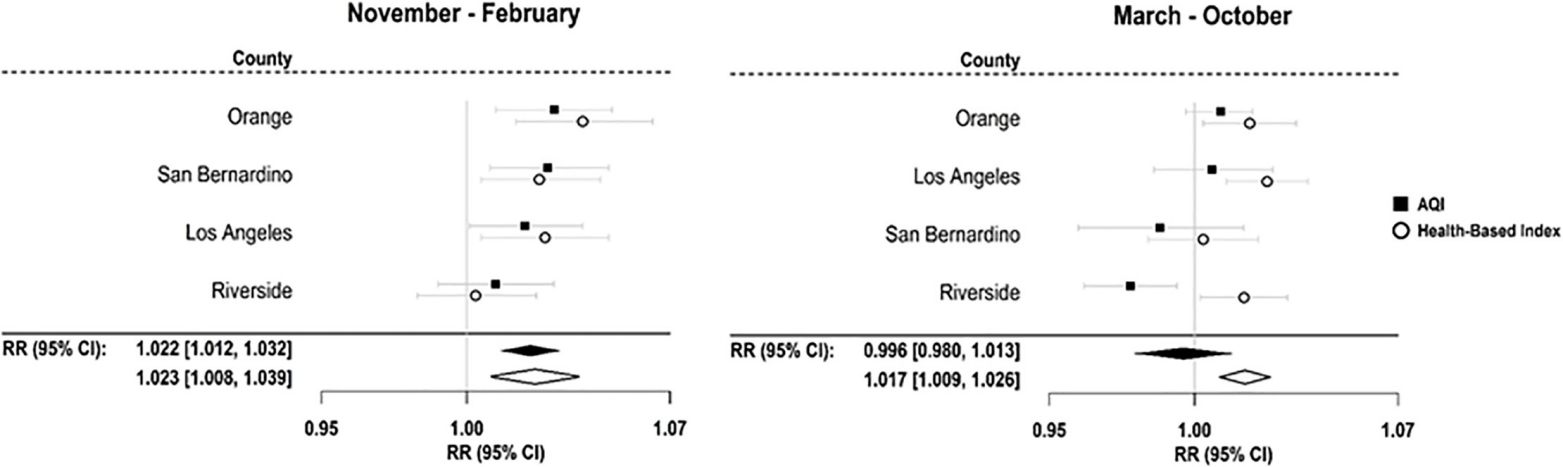
Results of an applied policy analysis showing relative risks of respiratory ED visits for health-based index vs. AQI in the Southcoast Air Quality Management District in Southern California from 2012–2014. Relative risks are presented per IQR increase of the index value. Abbreviations: AQI, air quality index; IQR, inter-quartile range; RR, relative risk.

As shown in Fig 4, the observed associations for both AQI values and health-based index values are nearly identical during the cooler months in the Southcoast Air Quality Management District, with significant associations observed in three of the four investigated counties for both indices. However, during the warmer season, the health-based index values were significantly associated with respiratory morbidity in 3 of the 4 counties during the warmer months, while the AQI was not positively associated with respiratory morbidity in any of the four counties and was even observed to be significantly, negatively associated with respiratory morbidity in one county. The pooled estimate of the association between the heath-based index and respiratory ED visits stays consistently significant throughout the year, with a combined RR of 1.02 (95% CI: 1.01, 1.04) during the cooler season and a combined RR of 1.02 (95% CI: 1.01, 1.03) during the warmer season. No such positive association was observed for the AQI, which had a combined RR of 1.00 (95% CI: 0.98, 1.01).

## Discussion

Even in circumstances when the AQI’s underlying pollutants are associated with respiratory health risks, there is no guarantee the index itself will be. Very few studies have assessed this association between AQI values and health outcomes in the US. In a review by Chen and Copes 2013 [29], only two studies were found meeting these criteria and few new studies of this nature have been identified since the time of the review. And while the studies available investigated the relationship between the AQI and specific health outcomes in their cohorts of interest, both used the AQI as a representation of true pollution rather than looking at whether AQI values are associated with population-level respiratory health outcomes, as done in the present analysis. This also does not include studies that use AQI categories to report results while directly assessing the associations of AQI values with adverse health outcomes [30]. However, the results of these other studies still offer insight into the conditions necessary for an effective AQI.

In one study, Balluz et al. 2007 [31] assessed ischemic heart disease data from the Behavioral Risk Factor Surveillance System (BRFSS) and the association with annual PM_2.5_ AQI values from the EPA’s Air Quality System (AQS) database across 51 U.S. counties. Adjusting for covariates, they found that counties with higher PM_2.5_-driven index values were significantly associated with ischemic heart disease across the country. In this study, associations did not vary significantly across different annual quarters. In contrast, investigating a narrower cohort, Letz and Quinn 2005 [32] did not observe significant associations between AQI levels and morbidity. Specifically, they found that among basic military trainees in San Antonio, Texas, the number of daily ED visits for asthma during the ozone season was not associated with either PM_2.5_- or O_3_-determined AQI levels. This suggests regional and seasonal variability could play a role in the AQI’s association with health risk.

In the present analysis, AQI values were found to be positively associated with respiratory risk across a large number of study counties, particularly during the cooler months of the year. This outcome suggests that in addition to its intended design to communicate extreme air quality events to the public, the AQI can function as a daily risk communication tool to inform behavior modification decisions—but only at certain times and locations.

Results show that in California, the AQI was most effective at predicting respiratory risk when index values are most highly correlated with PM_2.5_ (see Table 3). In all three regions, the correlations between AQI values and PM_2.5_ concentrations are over 0.89 during the cooler months of the year. Thus, during this season, the AQI essentially represents the population level health risks of daily PM_2.5_ concentrations which was observed to be positively associated with health outcomes in all three regions.

It is unsurprising to see that AQI values are not reflective of population-level morbidity in the San Joaquin region during the warmer months of the year given that none of the individual pollutants used to calculate the AQI were observed to have significant positive associations. This result does not necessarily mean that air pollution is not an important risk factor for respiratory health in this region, but more likely suggests that pollution levels measured at central site monitors are not reflecting the individual exposures responsible for excess morbidity. Improved air pollution monitoring, which better reflects population-level exposures, may be needed in these and other similar locations before effective risk communication is possible. This could include increasing the density of monitors, improving the placement of monitors, or in using alterative modern techniques that may better reflect distribution of the spatial distribution of pollutants [33].

While a positive association between at least some of the individual air pollutants and health outcomes is an essential condition for the AQI to reflect population-level health risks, this condition alone does not guarantee that AQI values will be significantly associated with health outcomes. For instance, during the warmer season, both Southern California and San Francisco Bay showed positive significant associations between respiratory ED visits and the individual pollutants PM_2.5_ and NO_2_; yet only daily AQI values in the San Francisco Bay region were significantly associated with respiratory ED visits (Fig 2). There are several potential characteristics of the study regions that may be important factors contributing to this result including: the high correlation of AQI values and O_3_ in the Southern California region (Table 3) and the higher concentrations and variability of multiple pollutants in Southern California as compared to the San Francisco Bay area (Table 2). A broader investigation involving a larger number of locations will be needed to definitively determine whether it is the unequal health risks of various pollutants on a per unit basis [34], the inability of the AQI to account for the combined health risks of multiple pollutants [35], or some other yet to be identified reason that best explains why the AQI values are not associated with population-level risks in times and locations in which positive associations are observed for the underlying pollutants.

The health-based index used in this study was created to determine if a multi-pollutant index constructed based on health studies, rather than regulatory levels, could better predict health risks in situations where AQI values are not associated with population-level health outcomes as part of an applied policy analysis. Results from an analysis of the four Southcoast Air Quality Management District counties showed that these generic health-based index values not only predicted health risks during the cooler months in a similar manner to AQI values, but also consistently predicted respiratory morbidity during the warmer months when the AQI could not (Fig 4).

The generic health-based index used in this analysis was based on coefficients derived from a meta-analysis of nationally representative studies as opposed to creating a region-specific health index using coefficients derived specifically from this region. Even though a tailor-made index using region-specific coefficients may provide even better results, the goal of this study was not to create an optimal index, but rather to assess whether a multi-pollutant, health-based approach could generally remedy the risk communication limitations of the AQI. Additional analysis would need to be completed before considering alternative index formulations in other locations, and it shouldn’t be assumed that just because a health-based index was observed to be better approach in one area that it will be the correct approach elsewhere.

The findings of this study have important implications for both practitioners and patients. The U.S. EPA recommends that medical pratitioners advise patients to use the AQI to inform behavior modification decisions [36] despite a lack of scientific evidence that typically accompanies clinical practice recommendations [15, 37]. The results of this study demonstrate that while AQI values are associated with population-level health risks in some locations for part of the year, the index may not reliably function as a risk communication tool when the monitored concentrations of the underlying pollutants are not associated with population-level health risks (as was the case in the San Joaquin Region during the warmer months of the year in this study). In these circumstances, it is advisable to consider efforts to improve exposure estimates used in generating index values to better reflect population-level exposures. Should well-executed exposure assessments still produce poor associations between pollutant concentrations and AQI values, it is possible that these associations simply do not hold true under the given situation. Alternatively, in circumstances where the underlying pollutants are associated with health outcomes but AQI values are not (such as Southern California during the warmer months of the year) it may be advisable to consider alternative approaches to the AQI such as a health-based index. Overall, the inconsistent ability of the AQI to reliably reflect daily health risks may explain why patients in some locales have reported relying on personal observations of outdoor conditions as much as air quality indices to inform behavior modification decisions [38].

This analysis was unable to assess differential impacts among various subpopulations. In addition to any increased susceptibility to the adverse effects of outdoor air pollution among individuals with low socio-economic indicators [39] there are also important equity issues as it relates to the ability to access and respond to risk information presented via the AQI [40]. Even in circumstances in which the AQI is predictive of health risks among susceptible individuals, if those same individuals are unable of these advisories or based on personal circumstances cannot modify their behaviors in ways to minimize their exposures to outdoor pollution, then the index may have limited functionality to protect health. Additionally, this study had insufficient power to subset results according to different AQI value groupings, and as such its associations with health outcomes at very low or very high pollution levels remains unclear. However, the authors were primarily concerned with characterizing the efficacy of the AQI at the most common, moderate levels, which has been well defined in this analysis.

Further efforts to evaluate and improve risk communication indices are warranted given the potential health benefits of individual behavior modification to reduce exposure to outdoor air pollution. Regardless of which index design is used to communicate monitored and predicted air pollution concentrations to the general public, the utilized approach should be tested and evaluated to ensure that it is reflective of the relevant health outcomes of interest.

## Conclusion

The use of air quality indices to communicate health risks associated with air pollution exposure is widespread in the United States and abroad, with vulnerable populations becoming increasingly reliant on these reports to avoid harmful pollution exposure. While the AQI was not specifically designed to function as a risk communication tool, it may be generally able to fulfill that objective as long as AQI values are highly correlated with a single pollutant (particularly PM_2.5_). However, in locations and during times with more complex air mixtures, AQI values may not fully capture population-level respiratory health risks, even when positive associations are observed for the underlying pollutants. In these and similar circumstances, it may be advisable for local air quality agencies to explore alternative risk communication approaches so that the public receives accurate health information.

## Supporting information

S1 Appendix Methods used to derive the coefficients used in the development of a generic, health-based index. (DOCX)

S1 Table Summary of studies used in meta-analysis to derive beta values and corresponding risk ratios for the health-based index. Final coefficients used in the creation of the health-based index were derived using random effects pooling.
(DOCX)

S2 Table Total population numbers, counts of respiratory ED visits, and mean (standard deviation) of daily AQI, 1-hour NO_2_, 8-hour ozone, and 24-hour PM_2.5_ concentrations, by season and county, between 2012–2014. Pollution units are ppb for NO_2_ and O_3_ and μg/m^3^ for PM_2.5_.
(DOCX)

S3 Table Relative risks of respiratory ED visits for ambient air pollutants at individual lag days 0–5, by season and region, from 2012–2014. Relative risks are presented per interquartile increase in pollutant concentrations.
(DOCX)

S4 Table Adjusted R^2^ values comparing observed vs. fitted values for each predictor variable by location and season. (DOCX)

S5 Table Post-hoc analysis of the impact of socioeconomic status on the relative risks of ED admission from increases in AQI and health-based index values. (DOCX)
