# Supplementary material for: Evaluating the U.S. Air Quality Index as a risk communication tool: Comparing associations of index values with respiratory morbidity among adults in California
Source: PLoS One. 2020 Nov 17;15(11):e0242031. doi: 10.1371/journal.pone.0242031 (PMC7671501; doi:10.1371/journal.pone.0242031)
Supplement: S1 Appendix — (DOCX) [file pone.0242031.s001.docx]

**S1 Appendix. Methods used to derive the coefficients used in the development of a generic, health-based index.**

The basic approach used to create the health-based air quality index used in the present study is to determine the additive effects of pooled coefficients calculated from a random effects meta-analysis of health-based studies of PM_2.5_, O_3_, and NO_2_ with respiratory morbidity in the US. The coefficients used in the creation of this index were derived following a systematic literature search that was conducted using Ovid Medline, Embase, Web of Science, Cumulative Index of Nursing and Allied Health, and Cochrane Library to identify English language studies published up until November 2017 that assessed associations between ambient air pollution and respiratory morbidity in the United States. Any studies without extractable data or that did not provide coefficients for respiratory outcomes were excluded. A list of the included studies and coefficients used in the meta-analysis can be found in Table E1 in the Online Data Supplement.

The final effect estimates derived from the random effects meta-analyses for constructing the health-based index were 1.024 (95% CI: 1.017-1.027) per 10 μg/m^3^ of PM_2.5_, 1.008 (95% CI: 1.004-1.013) per 10 ppb of O_3_, and 1.008 (95% CI: 1.004-1.013) per 10 ppb of NO_2_. It is not intended that these coefficients be used in the development of future air quality indices, but rather they were constructed to evaluate how a generically constructed health-based index might compare to the AQI in its ability to predict population-level respiratory health risks. It is noteworthy that the health-based index evaluated in this study is not location-specific to California, and as a result is not optimized to the study locations evaluated in this study. The creation of future health-based air quality indices would benefit from a more precise and thorough development of location-specific coefficients.

The equation used to calculate daily index values was taken from a paper by Perlmutt and Cromar (2019), shown below:

Health-Based Index = $\frac{10}{M}\sum_{t=1\ldots p} \ln[100\left( e^{\beta_{i}X_{it}}-1 \right)]$

where *M* is the maximum index value over the study period, *X_it_* is the daily concentration for pollutant *i* on day *t*, and *β* is the coefficient for pollutant *i* derived from the meta-analysis described above [1].

**Reference**

1. Perlmutt LD, Cromar KR. Comparing associations of respiratory risk for the EPA Air Quality Index and health-based air quality indices. Atmospheric Environment. 2019;202:1-7. doi: 10.1016/j.atmosenv.2019.01.011.
