# Supplementary material for: Evaluating the U.S. Air Quality Index as a risk communication tool: Comparing associations of index values with respiratory morbidity among adults in California
Source: PLoS One. 2020 Nov 17;15(11):e0242031. doi: 10.1371/journal.pone.0242031 (PMC7671501; doi:10.1371/journal.pone.0242031)
Supplement: S1 Table — Final coefficients used in the creation of the health-based index were derived using random effects pooling. (DOCX) [file pone.0242031.s002.docx]

**S1 Table. Summary of studies used in meta-analysis to derive beta values and corresponding risk ratios for the health-based index.** Final coefficients used in the creation of the health-based index were derived using random effects pooling.

| **O_3_** | **RR (95% CI)*** | **Study Type** | **Beta Value^†^** | **SE^†^** |
| --- | --- | --- | --- | --- |
| Pooled estimate | 1.008 (1.004, 1.013) | RE | 0.007696 | 0.0021 |
| Sheppard et al, 1999 [1] | 1.06 (1.02, 1.11) | TS | 0.002469 | 0.0009459 |
| Peel et al, 2005 [2] | 1.024 (1.008, 1.039) | TS | 0.0007187 | 0.0002542 |
| Wilson et al, 2005a [3] | 0.98 (0.950, 1.020) | TS | 0.0009486 | 0.0003091 |
| Wilson et al, 2005b [3] | 0.99 (0.970, 1.010) | TS | 0.002955 | 0.001752 |
| Sinclair et al, 2010a [4] | 0.979 (0.887, 1.081) | TS | -0.003355 | 0.002716 |
| Sinclair et al, 2010b [4] | 0.983 (0.914, 1.056) | TS | 0.003826 | 0.003557 |
| Sinclair et al, 2010c [4] | 1.024 (0.995, 1.054) | TS | 0.002913 | 0.001079 |
| Sinclair et al, 2010d [4] | 1.022 (0.987, 1.059) | TS | -0.0008839 | 0.002101 |
| Strickland et al, 2010 [5] | 1.062 (1.031, 1.093) | CC | -0.0007141 | 0.001534 |
| Glad et al, 2012 [6] | 1.025 (1.006, 1.044) | CC | 0.0009878 | 0.0006120 |
| Rodopoulou et al, 2014a [7] | 0.967 (0.917, 1.02) | TS | 0.0009064 | 0.0007481 |
| Rodopoulou et al, 2014b [7] | 1.039 (0.969, 1.114) | TS | 0.002060 | 0.0005101 |
| Wendt et al, 2014a [8] | 1.05 (1.02, 1.08) | CC | 0.004879 | 0.001458 |
| Wendt et al, 2014b [8] | 1.02 (0.97, 1.06) | CC | 0.0019805 | 0.002263 |
| Pride et al, 2015 [9] | 1.03 (0.999, 1.07) | CC | -0.001 | 0.0008977 |
| Winquist et al, 2015b [10] | 1.048 (1.013, 1.084) | TS | -0.001 | 0.001026 |
| Katasouyanni et al, 2009 [11] | 1.0036 (1.0011, 1.0061) | TS | 0.001660 | 0.0006117 |
|  |  |  |  |  |
| **PM_2.5_** | **RR (95% CI)*** | **Study Type** | **Beta Value^†^** | **SE^†^** |
| Pooled estimate | 1.022 (1.017, 1.027) | RE | 0.002151 | 0.0024 |
| Sheppard et al, 1999 [1] | 1.04 (1.01, 1.07) | TS | 0.003324 | 0.0012475 |
| Peel et al, 2005 [2] | 1.016 (0.997, 1.035) | TS | 0.001587 | 0.0009542 |
| Slaughter et al, 2005a [12] | 1.01 (0.98, 1.04) | TS | 0.000995 | 0.001516 |
| Slaughter et al, 2005b [12] | 0.98 (0.94, 1.01) | TS | -0.002020 | 0.0018322 |
| Mar et al, 2009 [13] | 1.04 (1.01, 1.07) | TS | 0.005603 | 0.002103 |
| Sinclair et al, 2010a [4] | 1.003 (0.932, 1.079) | TS | 0.0003288 | 0.004101 |
| Sinclair et al, 2010b [4] | 1.001 (0.951, 1.053) | TS | 0.0001097 | 0.002853 |
| Sinclair et al, 2010c [4] | 1.015 (0.99, 1.04) | TS | 0.001634 | 0.001380 |
| Sinclair et al, 2010d [4] | 1.007 (0.988, 1.027) | TS | 0.0007657 | 0.001084 |
| Strickland et al, 2010 [5] | 1.02 (1.002, 1.039) | CC | 0.002129 | 0.0009946 |
| Li et al, 2011 [14] | 1.012 (0.985, 1.04) | TS | 0.001302 | 0.001513 |
| Glad et al, 2012 [6] | 1.036 (1.001, 1.073) | CC | 0.003537 | 0.0017719 |
| Kim et al, 2012 [15] | 0.998 (0.974, 1.024) | TS | -0.0004410 | 0.002813 |
| Gleason et al, 2014 [16] | 1.03 (1.02, 1.04) | CC | 0.003433 | 0.0005753 |
| Wendt et al, 2014a [8] | 1.12 (1.03, 1.22) | CC | 0.01133 | 0.004319 |
| Wendt et al, 2014b [8] | 1.1 (0.99, 1.22) | CC | 0.009531 | 0.005329 |
| Winquist et al, 2015a [10] | 0.995 (0.98, 1.011) | TS | -0.0004524 | 0.0007170 |
| Winquist et al, 2015b [10] | 1.037 (1.006, 1.069) | TS | 0.003279 | 0.001398 |
| Ostro et al, 2009 [17] | 1.041 (1.018, 1.064) | TS | 0.002752 | 0.0007722 |
| Zanobetti et al, 2009 [18] | 1.0207 (1.012, 1.0295) | TS | 0.002049 | 0.0004374 |
|  |  |  |  |  |
| **NO_2_** | **RR (95% CI)*** | **Study Type** | **Beta Value^†^** | **SE^†^** |
| Pooled estimate | 1.008 (1.004, 1.013) | RE | 0.007696 | 0.0021 |
| Ito et al, 2007 [19] | 1.14 (1.09, 1.19) | TS | 0.005460 | 0.0009330 |
| Tolbert et al, 2007 [20] | 1.015 (1.004, 1.025) | TS | 0.000647 | 0.0002296 |
| Sinclair et al, 2010a [4] | 1.038 (0.971, 1.11) | TS | 0.002086 | 0.001909 |
| Sinclair et al, 2010b [4] | 0.988 (0.948, 1.03) | TS | -0.0006752 | 0.001184 |
| Sinclair et al, 2010c [4] | 1.011 (0.939, 1.088) | TS | 0.0006119 | 0.002101 |
| Sinclair et al, 2010d [4] | 0.989 (0.942, 1.038) | TS | -0.0006186 | 0.001385 |
| Sinclair et al, 2010e [4] | 1.022 (0.996, 1.049) | TS | 0.001217 | 0.0007397 |
| Sinclair et al, 2010f [4] | 1.003 (0.984, 1.022) | TS | 0.000168 | 0.0005406 |
| Strickland et al, 2010 [5] | 1.036 (1.018, 1.055) | CC | 0.002742 | 0.0007060 |
| Grineski et al, 2011[21] | 1.03 (0.98, 1.09) | CC | 0.002956 | 0.002714 |
| Li et al, 2011 [14] | 0.972 (0.93, 1.016) | TS | -0.002943 | 0.002338 |
| Wendt et al, 2014a [8] | 1.07 (1.03, 1.11) | CC | 0.006766 | 0.001908 |
| Wendt et al, 2014b [8] | 1.01 (0.98, 1.05) | CC | 0.0009950 | 0.001760 |
| Winquist et al, 2015a [10] | 1.009 (0.993, 1.027) | TS | 0.0007466 | 0.0007157 |
| Winquist et al, 2015b [10] | 1.048 (1.015, 1.083) | TS | 0.003907 | 0.001379 |

Abbreviations: CC, case-crossover; RE, random effects; SE, standard error; TS, time series

*Relative risks standardized to a pollutant increment of 10 ppb for O_3_ and NO_2_ and an increment of 10 μg/m^3^ for PM_2.5_.

**^†^**Beta values and standard errors are representative of a one-unit increase in the associated pollutant.

**References**

1. Sheppard L, Levy D, Norris G, Larson T, JQ K. Effects of ambient air pollution on nonelderly asthma hospital admissions in Seattle, Washington, 1987-1994. Epidemiology 1999; 10: 23-30.

2. Peel JL, Tolbert PE, Klein M, Metzger KB, Flanders WD, Todd K, Mulholland JA, Ryan PB, Frumkin H. Ambient Air Pollution and Respiratory Emergency Department Visits. Epidemiology 2005; 16: 164-174.

3. Wilson AM, Wake CP, Kelly T, Salloway JC. Air pollution, weather, and respiratory emergency room visits in two northern New England cities: an ecological time-series study. Environ Res 2005; 97: 312-321.

4. Sinclair AH, Edgerton ES, Wyzga R, Tolsma D. A Two-Time-Period Comparison of the Effects of Ambient Air Pollution on Outpatient Visits for Acute Respiratory Illnesses. Journal of the Air & Waste Management Association 2010; 60: 163-175.

5. Strickland MJ, Darrow LA, Klein M, Flanders WD, Sarnat JA, Waller LA, Sarnat SE, Mulholland JA, Tolbert PE. Short-term associations between ambient air pollutants and pediatric asthma emergency department visits. Am J Respir Crit Care Med 2010; 182: 307-316.

6. Glad J, Brink L, Talbott E, Lee P, Xu X, Saul M, J R. The relationship of ambient ozone and PM(2.5) levels and asthma emergency department visits: possible influence of gender and ethnicity. Arch Environ Occup Health 2012; 67: 103-108.

7. Rodopoulou S, Chalbot MC, Samoli E, Dubois DW, San Filippo BD, Kavouras IG. Air pollution and hospital emergency room and admissions for cardiovascular and respiratory diseases in Dona Ana County, New Mexico. Environ Res 2014; 129: 39-46.

8. Wendt JK, Symanski E, Stock TH, Chan W, Du XL. Association of short-term increases in ambient air pollution and timing of initial asthma diagnosis among Medicaid-enrolled children in a metropolitan area. Environ Res 2014; 131: 50-58.

9. Pride KR, Peel JL, Robinson BF, Busacker A, Grandpre J, Bisgard KM, Yip FY, Murphy TD. Association of short-term exposure to ground-level ozone and respiratory outpatient clinic visits in a rural location - Sublette County, Wyoming, 2008-2011. Environ Res 2015; 137: 1-7.

10. Winquist A, Schauer JJ, Turner JR, Klein M, Sarnat SE. Impact of ambient fine particulate matter carbon measurement methods on observed associations with acute cardiorespiratory morbidity. J Expo Sci Environ Epidemiol 2015; 25: 215-221.

11. Katsouyanni K, Samet J, Anderson H, Atkinson R, Le Tertre A, Medina S, Samoli E, Touloumi G, Burnett R, Krewski D, Ramsay T, Dominici F, Peng R, Schwartz J, Zanobetti A, Committee HHR. Air pollution and health: a European and North American approach (APHENA). Res Rep Health Eff Inst 2009; 142: 5-90.

12. Slaughter JC, Kim E, Sheppard L, Sullivan JH, Larson TV, Claiborn C. Association between particulate matter and emergency room visits, hospital admissions and mortality in Spokane, Washington. J Expo Anal Environ Epidemiol 2005; 15: 153-159.

13. Mar T, Koenig J. Relationship between visits to emergency departments for asthma and ozone exposure in greater Seattle, Washington. Ann Allergy Asthma Immunol 2009; 103: 474-479.

14. Li S, Batterman S, Wasilevich E, Wahl R, Wirth J, Su FC, Mukherjee B. Association of daily asthma emergency department visits and hospital admissions with ambient air pollutants among the pediatric Medicaid population in Detroit: time-series and time-stratified case-crossover analyses with threshold effects. Environ Res 2011; 111: 1137-1147.

15. Kim SY, Peel JL, Hannigan MP, Dutton SJ, Sheppard L, Clark ML, Vedal S. The temporal lag structure of short-term associations of fine particulate matter chemical constituents and cardiovascular and respiratory hospitalizations. Environmental health perspectives 2012; 120: 1094-1099.

16. Gleason JA, Bielory L, Fagliano JA. Associations between ozone, PM2.5, and four pollen types on emergency department pediatric asthma events during the warm season in New Jersey: a case-crossover study. Environ Res 2014; 132: 421-429.

17. Ostro B, Roth L, Malig B, Marty M. The effects of fine particle components on respiratory hospital admissions in children. Environ Health Perspect 2009; 117: 475-480.

18. Zanobetti A, Franklin M, Koutrakis P, Schwartz J. Fine particulate air pollution and its components in association with cause-specific emergency admissions. Environ Health 2009; 8: 58.

19. Ito K, Thurston GD, Silverman RA. Characterization of PM2.5, gaseous pollutants, and meteorological interactions in the context of time-series health effects models. J Expo Sci Environ Epidemiol 2007; 17 Suppl 2: S45-60.

20. Tolbert PE, Klein M, Peel JL, Sarnat SE, Sarnat JA. Multipollutant modeling issues in a study of ambient air quality and emergency department visits in Atlanta. J Expo Sci Environ Epidemiol 2007; 17 Suppl 2: S29-35.

21. Grineski SE, Staniswalis JG, Bulathsinhala P, Peng Y, Gill TE. Hospital admissions for asthma and acute bronchitis in El Paso, Texas: do age, sex, and insurance status modify the effects of dust and low wind events? Environ Res 2011; 111: 1148-1155.
