# Supplementary material for: Evaluating the U.S. Air Quality Index as a risk communication tool: Comparing associations of index values with respiratory morbidity among adults in California
Source: PLoS One. 2020 Nov 17;15(11):e0242031. doi: 10.1371/journal.pone.0242031 (PMC7671501; doi:10.1371/journal.pone.0242031)
Supplement: S2 Table — Pollution units are ppb for NO2 and O3 and μg/m3 for PM2.5. (DOCX) [file pone.0242031.s003.docx]

**S2 Table. Total population numbers, counts of respiratory ED visits, and mean (standard deviation) of daily AQI, 1-hour NO_2_ , 8-hour ozone, and 24-hour PM_2.5_ concentrations, by season and county, between 2012-2014.** Pollution units are ppb for NO_2_ and O_3_ and μg/m^3^ for PM_2.5_.

| **Southern California** | | | | | | | |
| --- | --- | --- | --- | --- | --- | --- | --- |
| **County** | **Population** | **Season** | **ED Visits** | **AQI** | **NO_2_** | **Ozone** | **PM_2.5_** |
| Imperial | 174,528 | Year Round | 12,892 | 67 (31) | 31.5 (15.2) | 50.4 (12.7) | 10.9 (6.3) |
|  |  | Mar-Oct | 6,719 | 72 (31) | 28.8 (15.7) | 56 (11.2) | 10 (4.6) |
|  |  | Nov-Feb | 6,173 | 58 (29) | 37.1 (12.5) | 39 (6.7) | 12.6 (8.6) |
| Los Angeles | 9,818,605 | Year Round | 373,532 | 90 (35) | 40.1 (13) | 58.7 (17.1) | 11.4 (4.7) |
|  |  | Mar-Oct | 214,616 | 98 (37) | 36.9 (12.5) | 67 (14.2) | 11.1 (3.6) |
|  |  | Nov-Feb | 158,916 | 73 (22) | 46.5 (11.4) | 41.7 (6.8) | 12 (6.4) |
| Orange | 514,453 | Year Round | 88,822 | 59 (19) | 28.7 (14.5) | 45.3 (11.2) | 10.5 (5.6) |
|  |  | Mar-Oct | 51,384 | 59 (19) | 23.2 (12.6) | 49.4 (9.8) | 9.3 (3.6) |
|  |  | Nov-Feb | 37,438 | 60 (20) | 40 (11.3) | 36.9 (8.8) | 13 (8) |
| Riverside | 2,189,641 | Year Round | 97,072 | 99 (42) | 31.1 (11.6) | 62.4 (16.3) | 11.1 (5) |
|  |  | Mar-Oct | 54,872 | 112 (42) | 28.3 (11.5) | 70.4 (13.4) | 12.1 (4.4) |
|  |  | Nov-Feb | 42,200 | 71 (24) | 36.8 (9.7) | 46.1 (6.9) | 9.1 (5.6) |
| San Bernardino | 2,035,210 | Year Round | 110,201 | 95 (43) | 43.1 (13.2) | 63.8 (17.7) | 13.1 (5.3) |
|  |  | Mar-Oct | 63,380 | 111 (43) | 42.5 (14.3) | 72.3 (15.3) | 13.9 (5.1) |
|  |  | Nov-Feb | 46,821 | 62 (19) | 44.5 (10.4) | 46.6 (5.3) | 11.5 (5.5) |
| San Diego | 3,095,313 | Year Round | 99,776 | 70 (28) | 29.6 (13.4) | 53.3 (11) | 9.6 (3.7) |
|  |  | Mar-Oct | 58,153 | 73 (31) | 24.6 (11.7) | 58 (9.7) | 10 (3.5) |
|  |  | Nov-Feb | 41,623 | 64 (18) | 39.8 (10.6) | 43.9 (6.5) | 8.8 (4) |
| San Luis Obispo | 269,599 | Year Round | 11,166 | 61 (21) | 17.5 (9) | 50.3 (10.9) | 8.5 (3.8) |
|  |  | Mar-Oct | 6,582 | 66 (23) | 14.5 (7.3) | 54 (10.4) | 8.4 (3.9) |
|  |  | Nov-Feb | 4,584 | 52 (16) | 23.7 (8.8) | 42.9 (7.6) | 8.7 (3.8) |
| Santa Barbara | 423,895 | Year Round | 18,375 | 50 (14) | 20.4 (9.5) | 48.8 (7.7) | 8.2 (3.1) |
|  |  | Mar-Oct | 11,125 | 52 (16) | 16.4 (7.4) | 50.3 (8.2) | 8.6 (3.1) |
|  |  | Nov-Feb | 7,250 | 46 (10) | 28.5 (8.2) | 45.7 (5.5) | 7.4 (3) |
| Ventura | 823,318 | Year Round | 27,386 | 58 (22) | 21.3 (8.5) | 51 (11.2) | 8.7 (3.8) |
|  |  | Mar-Oct | 15,867 | 64 (22) | 18.9 (7.7) | 56 (9.6) | 10 (3.4) |
|  |  | Nov-Feb | 11,519 | 45 (12) | 26 (8.2) | 40.7 (6.4) | 6.1 (3.3) |
| All Regions | 19,344,562 | Year Round | 839,222 | 72 (34) | 29.3 (14.7) | 53.8 (14.6) | 10.2 (4.9) |
|  |  | Mar-Oct | 482,698 | 78 (37) | 26 (14.5) | 59.3 (14) | 10.4 (4.3) |
|  |  | Nov-Feb | 356,524 | 59 (22) | 35.9 (12.7) | 42.6 (7.5) | 9.7 (5.9) |
|  | | | | | | | |

| **San Joaquin Valley** | | | | | | | |
| --- | --- | --- | --- | --- | --- | --- | --- |
| **County** | **Population** | **Season** | **ED Visits** | **AQI** | **NO_2_** | **Ozone** | **PM_2.5_** |
| Fresno | 930,450 | Year Round | 36,853 | 90 (38) | 26.8 (13.5) | 55.7 (18.8) | 12.6 (9.5) |
|  |  | Mar-Oct | 21,085 | 91 (39) | 23 (11.7) | 65 (14.7) | 8.9 (3.5) |
|  |  | Nov-Feb | 15,768 | 89 (37) | 34.7 (13.5) | 36.5 (9.3) | 20.2 (12.7) |
| Kern | 839,631 | Year Round | 50,567 | 95 (38) | 27.6 (8.6) | 59.5 (15.1) | 12.9 (10.6) |
|  |  | Mar-Oct | 28,095 | 98 (37) | 26.3 (8.2) | 66.5 (12.4) | 9.5 (4.8) |
|  |  | Nov-Feb | 22,472 | 89 (40) | 30.3 (8.7) | 45.3 (8.8) | 19.8 (15) |
| Kings | 152,982 | Year Round | 19,336 | 77 (33) | 21.2 (10.9) | 50.3 (16.8) | 16.7 (15.1) |
|  |  | Mar-Oct | 10,260 | 74 (28) | 18.1 (10.2) | 58.3 (12.3) | 11 (5.5) |
|  |  | Nov-Feb | 9,076 | 83 (40) | 27.3 (9.5) | 31.5 (9.3) | 27.7 (20.8) |
| Madera | 150,865 | Year Round | 7,619 | 76 (32) | 15.6 (8.3) | 50.4 (15.7) | 16 (10.9) |
|  |  | Mar-Oct | 4,242 | 77 (32) | 12.4 (6.1) | 56.7 (11.9) | 12.2 (5.2) |
|  |  | Nov-Feb | 3,377 | 74 (32) | 20.8 (8.6) | 32.7 (10.7) | 23.3 (14.8) |
| Merced | 255,793 | Year Round | 15,397 | 64 (29) | 18 (9.6) | 48.8 (16.9) | 11.8 (9.4) |
|  |  | Mar-Oct | 8,633 | 64 (29) | 15.3 (8.2) | 55.6 (13.4) | 8.2 (4.1) |
|  |  | Nov-Feb | 6,764 | 65 (30) | 24 (9.7) | 29.8 (9.4) | 18.7 (12.3) |
| San Joaquin | 685,306 | Year Round | 46,591 | 64 (26) | 27.1 (11.8) | 45.7 (13.8) | 10.8 (8) |
|  |  | Mar-Oct | 26,370 | 60 (24) | 23.8 (11.1) | 51.9 (11.7) | 8.1 (3.9) |
|  |  | Nov-Feb | 20,221 | 72 (30) | 33.7 (10.4) | 33.4 (8.2) | 16.2 (10.9) |
| Stanislaus | 514,453 | Year Round | 38,513 | 70 (33) | 22.3 (10.9) | 42.8 (16.2) | 13.2 (11.5) |
|  |  | Mar-Oct | 21,207 | 67 (31) | 19.4 (10.1) | 51 (12.1) | 8.6 (4.5) |
|  |  | Nov-Feb | 17,306 | 76 (35) | 28.4 (10) | 26 (8.6) | 22.8 (15.1) |
| Tulare | 442,176 | Year Round | 18,839 | 89 (38) | 25.8 (12.3) | 56.2 (18.3) | 16.5 (12.8) |
|  |  | Mar-Oct | 10,505 | 92 (38) | 22.6 (11.5) | 65 (14.8) | 12.1 (4.9) |
|  |  | Nov-Feb | 8,334 | 84 (39) | 32.6 (11.2) | 38.1 (9) | 25.4 (18.2) |
| All Regions | 3,971,656 | Year Round | 233,715 | 78 (36) | 23.5 (11.7) | 51.3 (17.4) | 13.8 (11.4) |
|  |  | Mar-Oct | 130,397 | 78 (35) | 20.5 (10.8) | 58.9 (14.2) | 9.8 (4.9) |
|  |  | Nov-Feb | 103,318 | 79 (36) | 29.3 (11.3) | 34.5 (10.7) | 21.7 (15.7) |
|  | | | | | | | |
|  | | | | | | | |
|  | | | | | | | |

| **San Francisco Bay Area** | | | | | | | |
| --- | --- | --- | --- | --- | --- | --- | --- |
| **County** | **Population** | **Season** | **ED Visits** | **AQI** | **NO_2_** | **Ozone** | **PM_2.5_** |
| Alameda | 1,510,271 | Year Round | 84,780 | 48 (18) | 28.1 (11.3) | 37.5 (11.3) | 8.5 (5.4) |
|  |  | Mar-Oct | 50,150 | 47 (17) | 24.1 (10.1) | 41.5 (11) | 7.1 (3.5) |
|  |  | Nov-Feb | 34,630 | 52 (20) | 36.2 (9.1) | 29.4 (6.8) | 11.4 (7.2) |
| Contra Costa | 1,049,025 | Year Round | 52,061 | 47 (16) | 20.9 (9.6) | 39.3 (10.5) | 9.2 (4.8) |
|  |  | Mar-Oct | 30,705 | 46 (16) | 16.9 (7.9) | 43 (10) | 8 (3.4) |
|  |  | Nov-Feb | 21,356 | 48 (17) | 28.9 (7.5) | 31.8 (6.4) | 11.3 (6) |
| Monterey | 415,057 | Year Round | 19,721 | 39 (10) | 13.3 (9) | 38.2 (7.4) | 5.7 (2.7) |
|  |  | Mar-Oct | 11,674 | 40 (10) | 9.9 (7.3) | 39.8 (7.8) | 5.8 (2.9) |
|  |  | Nov-Feb | 8,047 | 36 (8) | 20.3 (8.1) | 34.9 (5.2) | 5.6 (2.4) |
| Napa | 136,484 | Year Round | 4,650 | 45 (15) | 17.1 (9.6) | 33.3 (9.1) | 11.3 (5.6) |
|  |  | Mar-Oct | 2,824 | 40 (12) | 12.9 (7.1) | 36 (8.5) | 8.9 (4.1) |
|  |  | Nov-Feb | 1,826 | 56 (15) | 25.7 (8.2) | 27.6 (7.8) | 15.8 (5.5) |
| San Mateo | 718,451 | Year Round | 30,629 | 40 (15) | 22.5 (10.8) | 29.6 (9.5) | 8.8 (5.5) |
|  |  | Mar-Oct | 17,715 | 37 (12) | 18.1 (8.6) | 32.2 (8.9) | 7.5 (3.8) |
|  |  | Nov-Feb | 12,914 | 45 (18) | 31.6 (9.2) | 24.2 (8.4) | 11.4 (7.2) |
| Santa Clara | 1,781,642 | Year Round | 58,466 | 49 (18) | 25.2 (12.1) | 32.7 (10.6) | 7.7 (3.9) |
|  |  | Mar-Oct | 33,937 | 46 (15) | 20.6 (9.8) | 36.9 (8.7) | 6.7 (3.4) |
|  |  | Nov-Feb | 24,529 | 53 (22) | 34.8 (10.6) | 23.6 (8.4) | 9.6 (4.1) |
| Solano | 413,344 | Year Round | 30,521 | 47 (17) | 18.8 (12) | 38.4 (10.3) | 9.7 (6) |
|  |  | Mar-Oct | 17,920 | 44 (15) | 13.5 (9.4) | 42.2 (9.6) | 8 (3.7) |
|  |  | Nov-Feb | 12,601 | 53 (20) | 29.6 (9.2) | 30.5 (6.8) | 13.1 (8) |
| Sonoma | 483,878 | Year Round | 19,400 | 39 (12) | 14.9 (9) | 34.6 (8.5) | 8.2 (4.6) |
|  |  | Mar-Oct | 11,365 | 37 (11) | 11.2 (6.6) | 36.5 (8.4) | 7.1 (3.6) |
|  |  | Nov-Feb | 8,035 | 43 (14) | 22.9 (8.2) | 30.9 (7.4) | 10.6 (5.5) |
| All Regions | 6,508,152 | Year Round | 300,228 | 44 (16) | 20.1 (11.5) | 35.5 (10.2) | 8.6 (5.1) |
|  |  | Mar-Oct | 176,290 | 42 (14) | 15.9 (9.6) | 38.6 (9.8) | 7.3 (3.7) |
|  |  | Nov-Feb | 123,938 | 48 (18) | 28.8 (10.2) | 29.2 (8) | 11.1 (6.6) |
