# Supplementary material for: Evaluating the U.S. Air Quality Index as a risk communication tool: Comparing associations of index values with respiratory morbidity among adults in California
Source: PLoS One. 2020 Nov 17;15(11):e0242031. doi: 10.1371/journal.pone.0242031 (PMC7671501; doi:10.1371/journal.pone.0242031)
Supplement: S3 Table — Relative risks are presented per interquartile increase in pollutant concentrations. (DOCX) [file pone.0242031.s004.docx]

**S3 Table. Relative risks of respiratory ED visits for ambient air pollutants at individual lag days 0-5, by season and region, from 2012-2014.** Relative risks are presented per interquartile increase in pollutant concentrations.

| **Southern California** | | | | |
| --- | --- | --- | --- | --- |
| **Season** | **Lag** | **PM_2.5_** | **Ozone** | **NO_2_** |
| March - October | Lag 0 | 1.012 (1.007 - 1.016) | 1.004 (1.000 - 1.009) | 1.002 (0.995 - 1.008) |
|  | Lag 1 | 1.011 (1.006 - 1.015) | 1.005 (1.000 - 1.010) | 1.014 (1.008 - 1.020) |
|  | Lag 2 | 1.006 (1.002 - 1.010) | 1.003 (0.998 - 1.007) | 1.021 (1.016 - 1.027) |
|  | Lag 3 | 1.003 (0.999 - 1.007) | 1.004 (1.000 - 1.009) | 1.022 (1.016 - 1.028) |
|  | Lag 4 | 0.998 (0.994 - 1.002) | 1.003 (0.998 - 1.008) | 1.014 (1.009 - 1.020) |
|  | Lag 5 | 0.994 (0.990 - 0.998) | 0.999 (0.995 - 1.004) | 1.008 (1.002 - 1.013) |
| November - February | Lag 0 | 1.021 (1.014 - 1.028) | 1.004 (1.000 - 1.009) | 0.997 (0.988 - 1.006) |
|  | Lag 1 | 1.020 (1.014 - 1.027) | 1.007 (1.002 - 1.012) | 1.000 (0.992 - 1.009) |
|  | Lag 2 | 1.013 (1.006 - 1.019) | 1.006 (1.002 - 1.011) | 1.010 (1.002 - 1.018) |
|  | Lag 3 | 1.006 (1.000 - 1.013) | 1.006 (1.001 - 1.011) | 1.011 (1.003 - 1.019) |
|  | Lag 4 | 1.003 (0.996 - 1.009) | 1.000 (0.996 - 1.005) | 1.006 (0.998 - 1.013) |
|  | Lag 5 | 0.999 (0.993 - 1.006) | 0.994 (0.989 - 0.999) | 1.003 (0.995 - 1.010) |
|  | | | | |
| **San Joaquin Valley** | | | | |
| **Season** | **Lag** | **PM_2.5_** | **Ozone** | **NO_2_** |
| March - October | Lag 0 | 0.998 (0.991 - 1.005) | 0.988 (0.974 - 1.003) | 0.984 (0.972 - 0.996) |
|  | Lag 1 | 1.000 (0.993 - 1.007) | 0.983 (0.968 - 0.998) | 0.992 (0.980 - 1.005) |
|  | Lag 2 | 1.001 (0.994 - 1.009) | 0.989 (0.974 - 1.003) | 0.987 (0.975 - 0.999) |
|  | Lag 3 | 1.003 (0.995 - 1.010) | 0.996 (0.981 - 1.010) | 0.997 (0.985 - 1.009) |
|  | Lag 4 | 1.009 (1.002 - 1.016) | 0.991 (0.977 - 1.004) | 1.012 (1.000 - 1.024) |
|  | Lag 5 | 0.999 (0.992 - 1.006) | 0.982 (0.969 - 0.994) | 1.011 (1.000 - 1.023) |
| November - February | Lag 0 | 1.026 (1.013 - 1.039) | 1.024 (1.014 - 1.034) | 1.008 (0.992 - 1.024) |
|  | Lag 1 | 1.039 (1.025 - 1.053) | 1.021 (1.011 - 1.030) | 1.010 (0.994 - 1.027) |
|  | Lag 2 | 1.037 (1.024 - 1.051) | 1.019 (1.010 - 1.028) | 1.021 (1.005 - 1.037) |
|  | Lag 3 | 1.035 (1.022 - 1.048) | 1.016 (1.007 - 1.025) | 1.028 (1.012 - 1.043) |
|  | Lag 4 | 1.013 (1.000 - 1.026) | 1.011 (1.002 - 1.019) | 1.020 (1.005 - 1.036) |
|  | Lag 5 | 1.007 (0.994 - 1.021) | 1.007 (0.998 - 1.016) | 1.005 (0.990 - 1.020) |
|  | | | | |
| **San Francisco Bay Area** | | | | |
| **Season** | **Lag** | **PM_2.5_** | **Ozone** | **NO_2_** |
| March - October | Lag 0 | 1.013 (1.003 - 1.022) | 1.009 (1.001 - 1.016) | 0.993 (0.982 - 1.004) |
|  | Lag 1 | 1.015 (1.006 - 1.024) | 1.012 (1.005 - 1.019) | 1.011 (1.001 - 1.022) |
|  | Lag 2 | 1.020 (1.011 - 1.030) | 1.012 (1.005 - 1.019) | 1.024 (1.014 - 1.034) |
|  | Lag 3 | 1.014 (1.005 - 1.024) | 1.014 (1.006 - 1.021) | 1.024 (1.014 - 1.034) |
|  | Lag 4 | 1.006 (0.997 - 1.015) | 1.007 (1.001 - 1.014) | 1.015 (1.006 - 1.025) |
|  | Lag 5 | 1.014 (1.005 - 1.023) | 1.007 (1.001 - 1.013) | 1.017 (1.008 - 1.026) |
| November - February | Lag 0 | 1.017 (1.006 - 1.028) | 0.994 (0.983 - 1.005) | 0.989 (0.978 - 1.001) |
|  | Lag 1 | 1.027 (1.016 - 1.039) | 1.008 (0.996 - 1.020) | 0.991 (0.980 - 1.002) |
|  | Lag 2 | 1.029 (1.018 - 1.041) | 1.009 (0.998 - 1.021) | 1.009 (0.998 - 1.020) |
|  | Lag 3 | 1.024 (1.013 - 1.035) | 1.008 (0.997 - 1.019) | 1.009 (0.998 - 1.020) |
|  | Lag 4 | 1.021 (1.010 - 1.032) | 1.010 (1.000 - 1.021) | 1.013 (1.003 - 1.024) |
|  | Lag 5 | 1.024 (1.013 - 1.035) | 1.004 (0.993 - 1.015) | 1.017 (1.007 - 1.028) |
