# Supplementary material for: Evaluating the U.S. Air Quality Index as a risk communication tool: Comparing associations of index values with respiratory morbidity among adults in California
Source: PLoS One. 2020 Nov 17;15(11):e0242031. doi: 10.1371/journal.pone.0242031 (PMC7671501; doi:10.1371/journal.pone.0242031)
Supplement: S4 Table — (DOCX) [file pone.0242031.s005.docx]

**S4 Table. Adjusted R^2^ values comparing observed vs. fitted values for each predictor variable by location and season.**

| **Season** | **Region** | **AQI** | **Health-based**  **Index** | **PM_2.5_** | **O_3_** | **NO_2_** |
| --- | --- | --- | --- | --- | --- | --- |
|  | Southern California | 0.94 | 0.94 | 0.94 | 0.94 | 0.94 |
| Year Round | San Joaquin Valley | 0.77 | 0.77 | 0.77 | 0.77 | 0.77 |
|  | San Francisco Bay Area | 0.84 | 0.84 | 0.84 | 0.84 | 0.84 |
|  | Southern California | 0.86 | 0.86 | 0.86 | 0.85 | 0.86 |
| Mar - Oct | San Joaquin Valley | 0.53 | 0.53 | 0.53 | 0.54 | 0.55 |
|  | SF Bay | 0.64 | 0.63 | 0.63 | 0.63 | 0.64 |
|  | Southern California | 0.85 | 0.85 | 0.85 | 0.85 | 0.85 |
| Nov - Feb | San Joaquin Valley | 0.61 | 0.62 | 0.61 | 0.61 | 0.58 |
|  | SF Bay | 0.77 | 0.76 | 0.77 | 0.76 | 0.76 |
