# Supplementary material for: Evaluating the U.S. Air Quality Index as a risk communication tool: Comparing associations of index values with respiratory morbidity among adults in California
Source: PLoS One. 2020 Nov 17;15(11):e0242031. doi: 10.1371/journal.pone.0242031 (PMC7671501; doi:10.1371/journal.pone.0242031)
Supplement: S5 Table — (DOCX) [file pone.0242031.s006.docx]

**S5 Table. Post-hoc analysis of the impact of socioeconomic status on the relative risks of ED admission from increases in AQI and health-based index values.**

| **Variable** | **Pollution Index** | **Beta** | **SE** | **p-value** |
| --- | --- | --- | --- | --- |
| Percent under poverty level | AQI | -0.92 | -1.13 | 0.42 |
|  | Health-based Index | -1.79 | 0.93 | 0.07 |
| Median household income | AQI | 0.25 | 0.26 | 0.35 |
|  | Health-based Index | 0.42 | 0.21 | 0.06 |

Abbreviations: AQI, Air Quality Index; ED, emergency department.

Note: Data on median household income and percent below the poverty line were obtained from the US Census Bureau [1] for each county and averaged over 2012-2014.

References

1. US Census Bureau. Small Area Income and Poverty Estimates (SAIPE): US Department of Commerce; 2020. Available from: https://www.census.gov/data-tools/demo/saipe/#/?map_geoSelector=aa_c&s_state=06&map_yearSelector=2013&s_year=2014,2013,2012.
